# Influence of food resources and prey egg age on the predatory efficiency of phytoseiid mites associated with cashew

**DOI:** 10.1007/s10493-026-01152-9

**Published:** 2026-06-12

**Authors:** Luana Lima Melo, Josiane P. De Alfaia, João M. R. Costa, Antônio de A. P. Neto, Debora B. Lima, José W. S. Melo

**Affiliations:** 1 Centro de Ciências Agrárias, Departamento de Fitotecnia, Universidade Federal do Ceará, Fortaleza, Brazil; 2 Programa de Pós-Graduação em Biologia Animal, Departamento de Zoologia, Laboratório de Acarologia, Universidade Federal de Pernambuco, Recife, Brazil

**Keywords:** *Amblyseius largoensis*, *Euseius concordis*, *Aleurodicus cocois*, Biological control, Giant whitefly, Nutrition, Natural enemies

## Abstract

Under field conditions, predator–prey interactions can be modulated by the diverse availability and suitability of food resources, which diet type may alter biological and behavioral parameters of predators. *Amblyseius largoensis* and *Euseius concordis* are phytoseiid predatory mites identified as potential biological control agents for the giant whitefly, *Aleurodicus cocois*, a major pest of cashew trees. In the present study, we investigated how exclusive and mixed diets composed of *Ricinus communis* pollen, *A. cocois* eggs, and *Tetranychus urticae* (used for comparison, as it is a standard prey in phytoseiid predation analysis) affect oviposition and predation by *A. largoensis* and *E. concordis*. In addition, we examined the effect of *A. cocois* egg age on consumption and feeding preference by these predators. Both species exhibited higher oviposition rates when fed exclusively on pollen, whereas diets composed solely of *T. urticae* resulted in the lowest values. The presence of pollen did not affect the predation rate of *A. cocois* eggs, indicating that the availability of this alternative resource does not compromise predatory efficiency. The age of *A. cocois* eggs offered as food did not affect predator oviposition; however, in both choice and no-choice tests, *A. largoensis* and *E. concordis* preferentially consumed older eggs (> 48 h). These results demonstrate that pollen availability increases reproductive potential without reducing predation capacity and that prey egg age influences consumption dynamics, providing ecologically relevant insights for management strategies that integrate the conservation of floral resources and the use of these phytoseiid mites in the biological control of *A. cocois* in cashew production systems.

## Introduction

Mites of the family Phytoseiidae are important natural enemies of arthropods in several agroecosystems and are considered biological control agents of agricultural pests (Moraes and Flechtmann 2008; Knapp et al. 2018; Tixier 2018; Vásquez et al. 2023). These mites exhibit generalist feeding habits, feeding on both arthropods (mites and insects) and plant-derived resources (e.g., pollen) (McMurtry and Croft 1997; McMurtry et al. 2013).

The generalist nature of these mites can positively or negatively affect biological control by modulating the interactions between predators and pests (van Rijn 2002; Janssen et al. 2003; van Maanen et al. 2012). High availability of food resources may result in a short-term negative effect on biological control, as predators can reach satiation rapidly, leading to reduced pest consumption (Abrams and Matsuda 1996; van Maanen et al. 2012; Bompard et al. 2013). However, the combination of prey and pollen can alter reproductive and behavioral parameters (e.g., predation), improving predator performance in pest population regulation (Messelink et al. 2008). For example, control of the whitefly *Bemisia tabaci* (Gennadius) (Hemiptera: Sternorrhyncha: Aleyrodidae), an important agricultural pest, was enhanced by providing pollen as food for the phytoseiid mite *Amblyseius swirskii* Athias-Henriot (Acari: Phytoseiidae) (Nomikou et al. 2009). In addition, the diversity of available resources can be an important factor by allowing predators to persist in agricultural production areas and regulate pest population densities in the long term (Holt 1977; van Rijn et al. 2002; Nomikou et al. 2009; Sarmento et al. 2011; Goleva and Zebitz 2013; Massaro et al. 2016; Tixier 2018).

*Amblyseius largoensis* (Muma) (Acari: Phytoseiidae) and *Euseius concordis* (Chant) (Acari: Phytoseiidae) are two predatory mites found on cashew plants (*Anacardium occidentale* L.), the latter being the predominant (Mendes et al. 2021). Both species act as important natural enemies of the giant whitefly, *Aleurodicus cocois* (Curtis) (Hemiptera: Sternorrhyncha: Aleyrodidae), a relevant pest of cashew crops, whose damage can be direct, through sap sucking, and indirect, through the dissemination of phytoviruses and facilitation of fungal infections (Byrne et al. 1990; Byrne and Bellows 1991; Mota et al. 2017). In Brazil, chemical control is the only recommended method to suppress the populations of *A. cocois* in cashew crops, but only three chemical groups (pyriproxyfen, pyrethroid and neonicotinoid) are recommended for controlling this pest (Agrofit 2026). This is a major problem, as repeated application of the same chemical molecule to control pests in agricultural environments leads to the selection of resistant populations. Biological control strategies offer a multifaceted approach to both preventing and mitigating the development of resistance (Liang et al. 2025). In this scenario, *Amblyseius largoensis* and *E. concordis* demonstrate potential for use in the control of *A. cocois* (Alfaia et al. 2018a, b).

*Amblyseius largoensis* and *E. concordis* are generalist mites that feed on a wide range of resources, such as small arthropods and pollen, and are classified as type III and type IV generalists, respectively (McMurtry and Croft 1997; Croft et al. 2004; McMurtry et al. 2013). Type III phytoseiids show good reproductive rates when feeding on prey (small arthropods), pollen, and nectar. Type IV generalists, although they also prey on small arthropods, are specialized pollen feeders and exhibit higher reproductive rates when this food source is available (Adar et al. 2012; Flechtmann and McMurtry 1992; Vangansbeke et al. 2023). The action of these phytoseiids occurs mainly through egg predation (Alfaia et al. 2018a). The age of the prey egg can interfere with the predator’s behavior, as demonstrated by the predatory mites: *Neoseiulus barkeri* (Hughes) (Acari: Phytoseiidae) preying on eggs of *T. urticae* (Momen and Hussein 2011), *(A) swirskii* preying on eggs of the whitefly *(B) tabaci* (Cavalcante et al. 2015), A. *swirskii* and *Neoseiulus californicus* (McGregor) (Mesostigmata: Phytoseiidae) preying on eggs of *T. urticae* (Akyazi et al. 2018), and *Blattisocius mali* (Oudemans) (Acari: Blattisociidae) preying on eggs of *Drosophila melanogaster* Meigen (Diptera: Drosophila) (Michalska et al. 2023). Therefore, analyzing predation on *A. cocois* eggs of different ages constitutes an important criterion for evaluating the regulatory capacity of these predators in pest control.

In agroecosystems, generalist predators such as *A. largoensis* and *E. concordis* can exploit a wide diversity of food resources and are not restricted solely to *A. cocois* eggs. Thus, in this study we evaluated how variation in food resources offered to these predatory mites can modify their oviposition and predation rates. To this end, we provided exclusive diets (a single food source) or mixed diets (a combination of different food sources) to females of *A. largoensis* and *E. concordis*. Exclusive diets consisted of *Tetranychus urticae* Koch (Acari: Tetranychidae), *A. cocois* eggs, or castor bean pollen (*Ricinus communis* L.), whereas mixed diets consisted of combinations of these food sources. Although not relevant to cashew trees, the mite *T. urticae* was used because it is widely used in tests with phytoseiid mites, being a standard prey in predation tests (Gravandian et al. 2022; Wang et al. 2024; Domingos et al. 2025). In addition, we evaluated the predation behavior of both predatory mite species when offered *A. cocois* eggs of different ages.

## Materials and methods

### Rearing of Amblyseius largoensis and Euseius concordis

Predatory mites were collected from cashew (*A. occidentale*) leaves obtained at the Federal University of Ceará, Pici Campus (3°44′19″ S, 38°34′11″ W). The mites were identified and established in rearing colonies consisting of arenas made of PVC sheets (10 × 10 cm) placed on foam discs (9 × 9 × 1 cm) inside plastic trays (16 cm in diameter). The edges of the PVC sheets were surrounded with hydrophilic cotton moistened with water to prevent mite escape. Cotton threads covered with a small PVC sheet (1 × 1 cm) were provided as oviposition sites. Fragments of jack bean leaves (*Canavalia ensiformis* L.) infested with T. *urticae*, 10% honey solution, and castor bean pollen (*Ricinus communis* L.) were offered as food sources. The colonies were maintained at 25 ± 2 °C, 70 ± 10% RH, and a 12-h photophase.

### Food sources

Cashew leaves (*A. occidentale*) infested with the cashew giant whitefly (*A. cocois*) were collected at the Federal University of Ceará, Pici Campus. The eggs of *A. cocois* used in the experiments were collected directly from these leaves, as this species is difficult to maintain under laboratory rearing conditions. The *T. urticae* population was obtained from the stock colony of the Laboratório de Manejo de Ácaros e Insetos (LAMAI). These tetranychids were maintained in arenas consisting of plastic trays (18 × 10 × 3.5 cm) containing moist polyethylene foam (17 × 9 × 1 cm), on which filter paper and a cotyledonary jack bean leaf (*C. ensiformis*) were placed. In each arena, the margins of the jack bean leaf were covered with hydrophilic cotton moistened with distilled water. Castor bean pollen (*R. communis*) was collected from recently opened inflorescences (approximately 24 h after opening), transferred to labeled Eppendorf tubes and stored under refrigeration until use. The pollen was used no more than one week after collection. Castor bean pollen was used because it is commonly employed in laboratory rearing of predatory mites (Marafeli et al. 2014; Soltaniyan et al. 2018). Honey was diluted to 10% in water, and a drop was deposited in the arena with the predators.

### Oviposition of Amblyseius largoensis and Euseius concordis

The effect of diet on predator oviposition was evaluated using the following treatments: (1) 0.2 mg of castor bean pollen; (2) 50 *A. cocois* eggs; (3) *T. urticae* only (two adult females introduced two days before the experiment to allow oviposition and the presence of different developmental stages (eggs), plus eight additional adult females added at the beginning of the experiment); (4) castor bean pollen + *T. urticae* (as described above); (5) 0.2 mg of castor bean pollen + 50 *A. cocois* eggs; and (6) *T. urticae* (as described above) + 50 *A. cocois* eggs. Females of both predator species were starved for a minimum period of four hours before the start of the experiment and then individualized in experimental units, with each female representing one replicate.

The experimental unit consisted of a Petri dish (8.5 cm in diameter × 1.0 cm in height) containing moist polyethylene foam (1 cm thick), on which filter paper and a fragment of cashew leaf (3 × 3 cm) or jack bean leaf (3 × 3 cm) (for the *T. urticae* treatment) were placed, according to the host plant required for maintaining each prey species. PVC sheets (1 × 1 cm) were placed for oviposition by the predator, and the edges of the leaves were wrapped, as described in the topic “Rearing of *Amblyseius largoensis* and *Euseius concordis*”. At the beginning of the treatments with *T. urticae*, eight additional adult females were added to maintain prey avaibility and avoid food limitation during the experimental period.

Predator oviposition rates were recorded every 24 h for four days; data from the first day were excluded due to possible interference from previous feeding. Under laboratory conditions, *A. cocois* eggs hatch in approximately seven days on average (Goiana et al. 2017); therefore, the evaluation at four days did not allow for hatching. Twenty replicates were performed for each treatment and predator species. At each evaluation, *T. urticae* females and *A. cocois* eggs were replenished to maintain the original density offered in each treatment, while castor bean pollen was replaced daily at the original amount of 0.2 mg.

### Addition of pollen and predation rate of Amblyseius largoensis and Euseius concordis on Aleurodicus cocois eggs

The influence of castor bean pollen on predation by both predator species was evaluated using *A. cocois* eggs as the target prey, since this whitefly is the pest of primary interest in this study. Thus, this assay was specifically designed to evaluate whether the availability of an alternative food resource affects predation on the focal pest species. Two treatments were used: (1) 50 *A. cocois* eggs only; and (2) 50 *A. cocois* eggs + 0.2 mg of castor bean pollen. For each predator species and treatment, 20 replicates were conducted, with each replicate consisting of a single adult female maintained in an individual experimental unit. Females of *A. largoensis* and *E. concordis* were starved for a minimum of four hours prior to the experiment to standardize hunger levels. The same experimental unit described above was used. Predation rates were evaluated over four-days period, with evaluations conducted every 24 h. Data from the first day were excluded from the analysis to minimize potential bias associated with prior feeding history and acclimation to the experimental conditions. At each evaluation, prey items were replenished to maintain the initial density established for each treatment.

### Predation preference of *Amblyseius largoensis* and *Euseius concordis* for *Aleurodicus cocois* eggs of different ages

Predator preference for eggs of different ages of the cashew giant whitefly was determined using choice and no-choice tests. Recently laid eggs and older eggs were used, where recent eggs were defined as those with up to 24 h of development, and old eggs as those with more than 48 h of development. These categories were established based on visual examination of eggs collected directly from naturally infested cashew leaves in the field. The leaves were transported to the laboratory immediately after collection, and egg age was classified according to coloration and visible developmental characteristics observed under a stereomicroscope. Eggs considered recent (≤ 24 h old) showed lighter coloration and no visible embryo development, whereas old eggs (> 48 h old) showed darker coloration and more advanced embryonic development. This classification allowed predation to be recorded separately for each age class.

In the no-choice test, 50 eggs of a single age class (recent or old) were transferred to each experimental unit and randomly distributed within the arena. A single predator female of *A. largoensis* or *E. concordis* was then introduced at the center of the arena to minimize positional bias in prey selection.

In the choice test, 30 recent eggs and 30 old eggs were transferred to each experimental unit and randomly offered to a single female of each predator species released at the experimental unit center.

Twenty replicates were conducted for each treatment. Predator females were starved for a minimum of four hours before the start of the experiment and then individualized in the experimental units, with each female corresponding to one replicate. The same experimental unit used in the previous bioassays was employed. Predation on each egg age class and oviposition levels were assessed after 24 h of confinement. As mentioned above, this time is not sufficient for the nymphs to hatch, even from old eggs.

### Statistical analysis

Oviposition data of predators fed exclusive diets or mixed diets consisting of *A. cocois* eggs, *T. urticae*, and *R. communis* pollen, as well as egg consumption data of predators fed only *A. cocois* eggs or *A. cocois* eggs plus *R. communis* pollen, were analyzed using Generalized Linear Models (GLM) with a quasipoisson distribution family. This distribution family was chosen due to overdispersion in the residuals of models fitted with a Poisson family. Model fit was assessed using an F test. The model’s response variable (Egg_number or Egg_consumption) represents the average of the three days of evaluation (both eggs laid and consumed, respectively).The number of eggs consumed and the number of eggs laid by females of both predator species when fed *A. cocois* eggs of different ages were analyzed using GLMs with a Poisson distribution family, as no overdispersion was observed in the model residuals. In this case, a chi-squared test was used to assess model fit. To compare the effect of different diets on the number of eggs of *E. concordis* and *A. largoens*, we used the lsmeans function from the emmeans package, employing Tukey’s test (α = 5%). Predator preference for *A. cocois* eggs of different ages was analyzed using a chi-squared test, comparing the frequency of consumption of eggs of different ages by the predators.

All analyses were performed in R Statistical Software (v4.1.2; R Core Team 2025). The DHARMa package was used to assess model residual diagnostics.

## Results

### Oviposition of Amblyseius largoensis and Euseius concordis

**Fig. 1:**
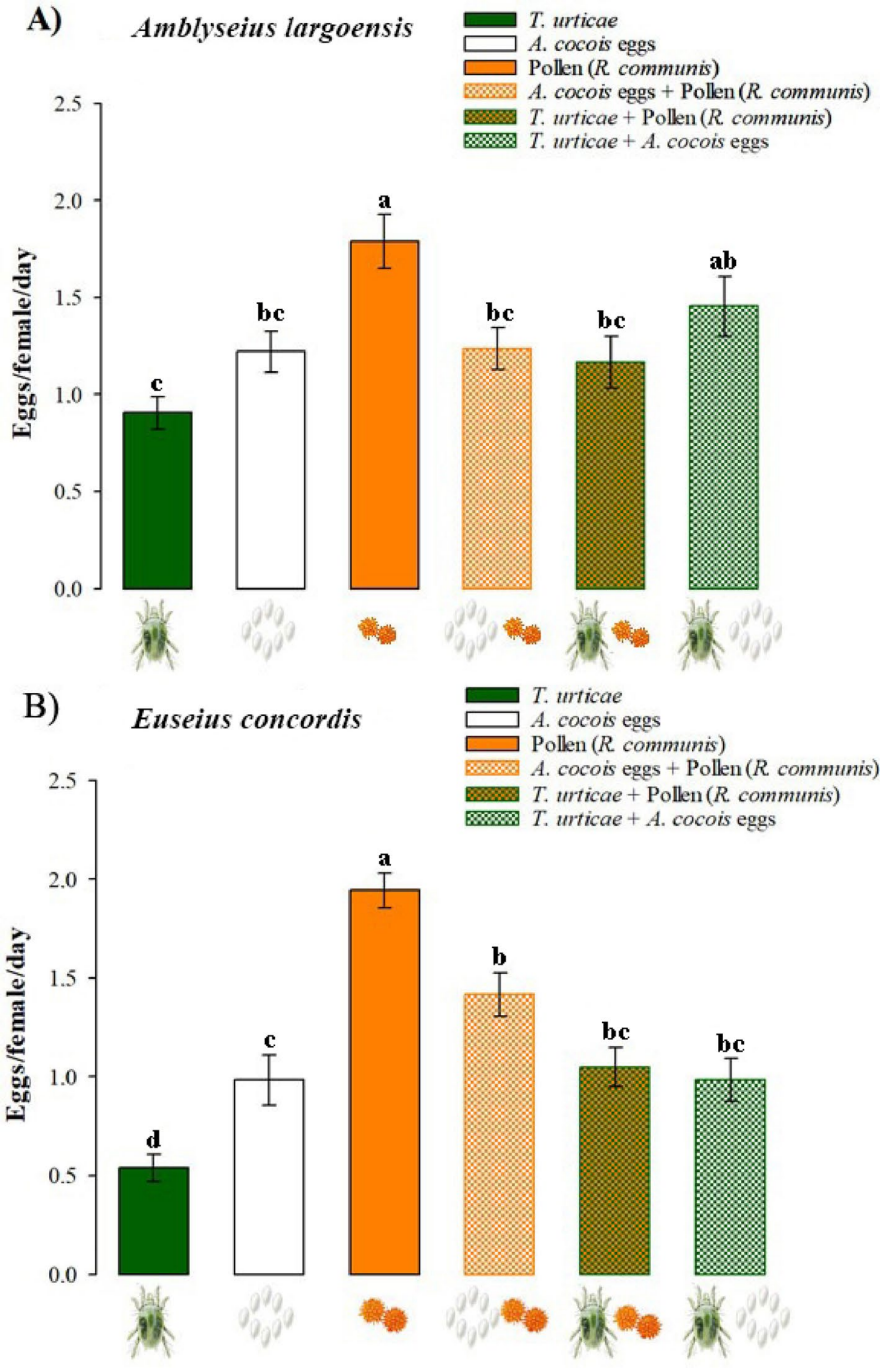
Daily oviposition (mean ± SE) by *A. largoensis* and *E. concordis* fed on *T. urticae*, *A. cocois* eggs, or pollen (*R. communis*). Different lowercase letters indicate a difference within each predator species by Tukey’s test (α = 5%)

Diet had a significant effect on the oviposition rate of both predators (*A. largoensis*, F₅,₁₂₄ = 5.84; *P* < 0.0001; and *E. concordis*, F₅,₁₂₄ = 20.85; *P* < 0.0001). For *A. largoensis*, the highest oviposition rate was observed on the diet composed exclusively of *R. communis* pollen, whereas the lowest rate occurred when females were fed exclusively on *T. urticae*. The remaining diets resulted in intermediate values; among these, the combination of *A. cocois* eggs with *T. urticae* did not differ from the pollen-only diet, whereas diets based on *A. cocois* eggs alone, pollen + *A. cocois* eggs, and pollen + *T. urticae* did not differ from the *T. urticae*-only diet (Fig. 1A).

For *E. concordis*, a similar pattern was observed, with the highest oviposition rate on the diet composed exclusively of *R. communis* pollen and the lowest rate when females were fed exclusively on *T. urticae*. The remaining diets yielded intermediate values, with a significant difference between the exclusive diet of *A. cocois* eggs and the combination of *A. cocois* eggs with *R. communis* pollen, the latter resulting in higher oviposition (Fig. 1B).

### Addition of pollen and predation rate of Amblyseius largoensis and Euseius concordis on Aleurodicus cocois eggs

**Fig. 2:**
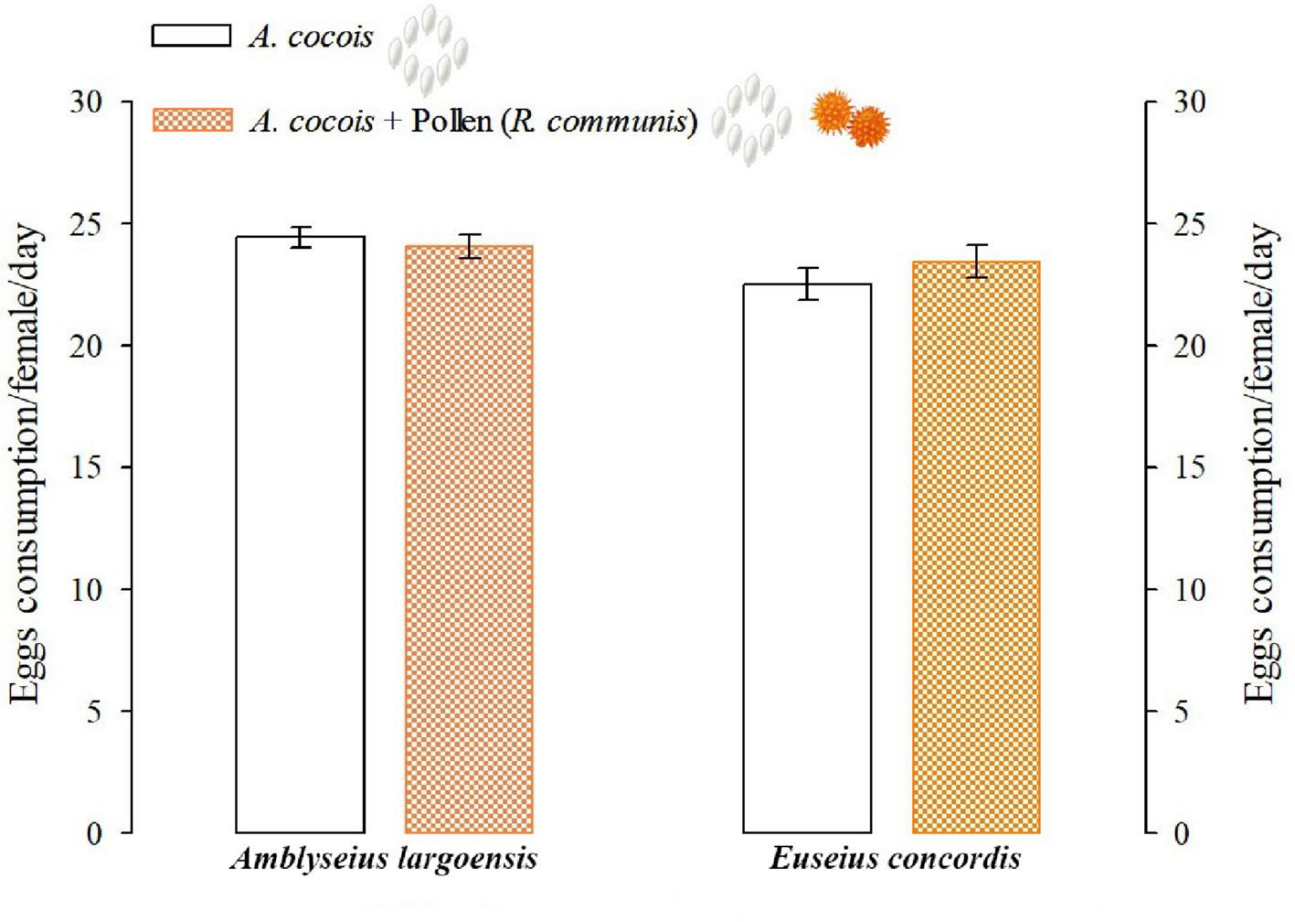
Daily predation (mean ± SE) of *A. cocois* eggs by *A. largoensis* and *E. concordis* in the presence and/or absence of *R. communis* pollen

The presence of *R. communis* pollen in the diet did not alter the predation rate on *A. cocois* eggs by the predatory mites, with no difference compared to predation when eggs were offered alone. This pattern was observed for both *A. largoensis* (GLM quasi-Poisson: F₁,₃₉ = 0.305; *P* = 0.584) and *E. concordis* (GLM quasi-Poisson: F₁,₄₃ = 0.999; *P* = 0.323) (Fig. 2).

### Predation preference of *Amblyseius largoensis* and *Euseius concordis* for *Aleurodicus cocois* eggs of different ages

**Fig. 3:**
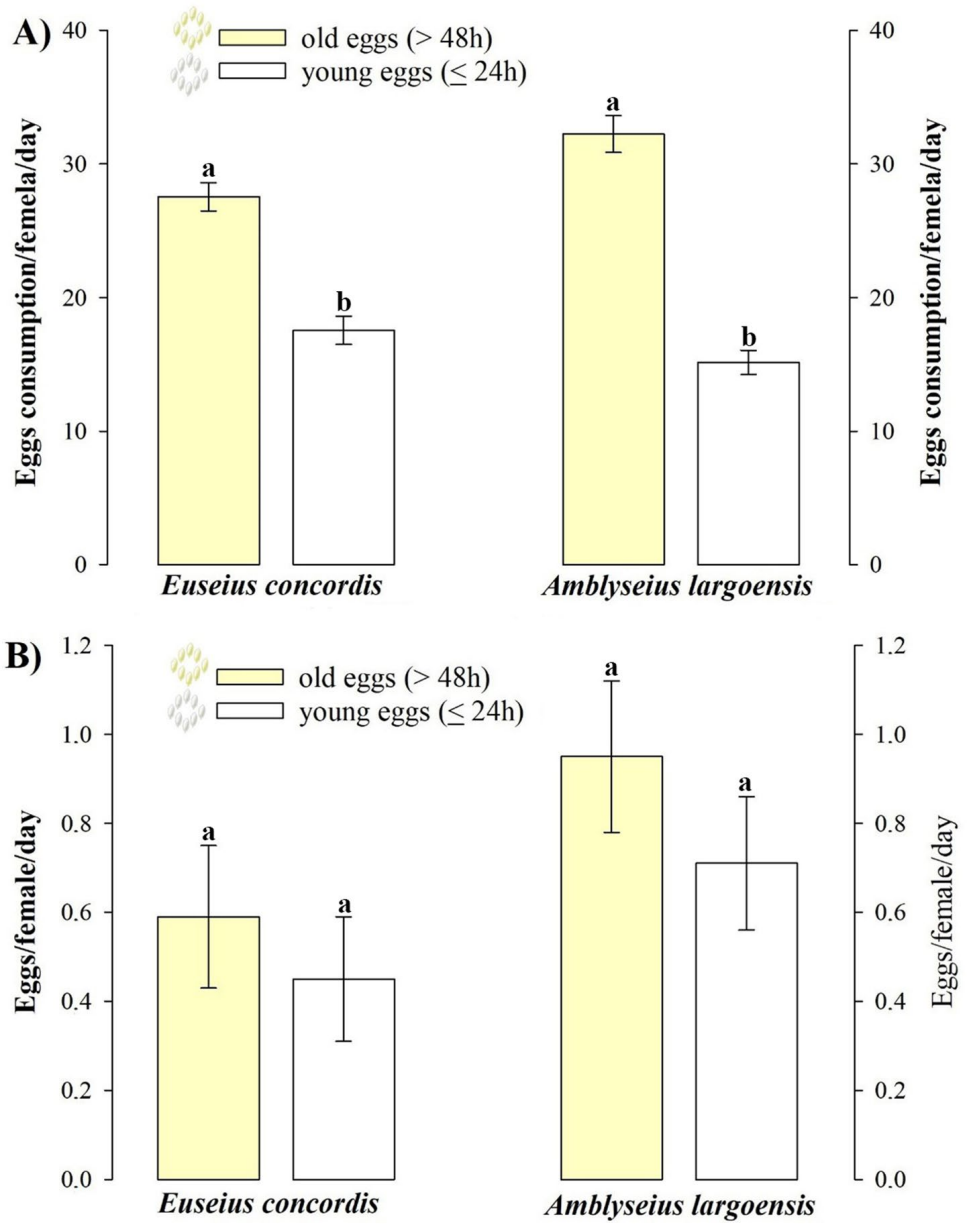
Mean consumption of *A. cocois* eggs (A) and mean oviposition (B) by *A. largoensis* and *E. concordis* when fed exclusively on *A. cocois* eggs of different ages (old or young eggs). Different lowercase letters indicate a difference within each predator species by Tukey’s test (α = 5%)

Predation on *A. cocois* eggs varied according to prey age, with higher consumption of older eggs (> 48 h) compared to younger eggs (≤ 24 h) by both *A. largoensis* (GLM Poisson: *χ²*_*(1)*_ = 132.5; *P* = 0.001) and *E. concordis* (GLM Poisson: *χ²*_*(1)*_ = 49.2; *P* = 0.001) (Fig. 3A). Mean consumption of older eggs was 32.2 eggs/female/day for *A. largoensis* and 27.5 eggs/female/day for *E. concordis*. Despite the higher predation on older eggs, egg age did not affect the oviposition rate of either predator. Although both predator species showed a tendency towards higher oviposition rates in older eggs, the difference was not statistically significant (*A. largoensis*, GLM Poisson: *χ²*_*(1)*_ = 0.717; *P* = 0.397; *E. concordis*, GLM Poisson: *χ²*_*(1)*_ = 0.392; *P* = 0.531) (Fig. 3B). For *A. largoensis*, mean oviposition rates were 0.71 and 0.95 eggs/female/day when fed young and old eggs, respectively, whereas for *E. concordis* they were 0.45 and 0.59 eggs/female/day.

**Fig. 4:**
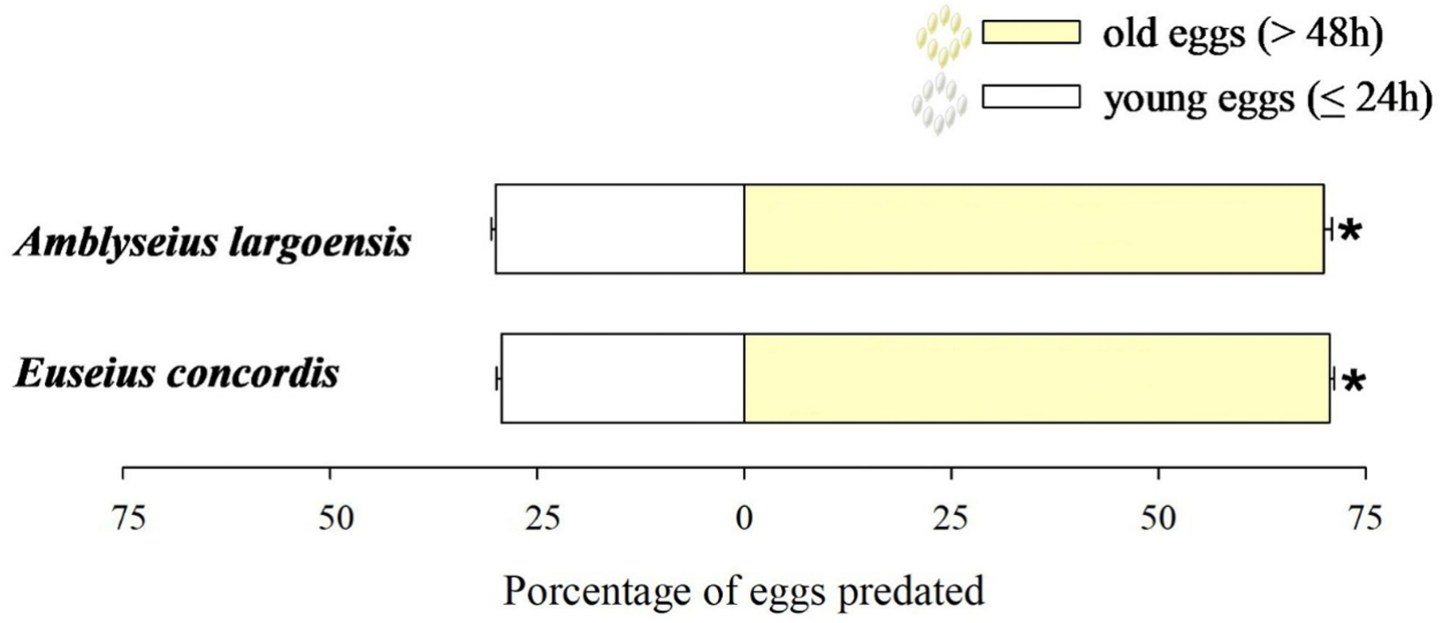
Choice test: consumption preference of old or young *A. cocois* eggs by *A. largoensis* and *E. concordis*. Asterisks indicate significant differences between treatments by Tukey’s test (α = 5%)

When given a choice between eggs of different ages, both predators showed a preference for older eggs (> 48 h), for *A. largoensis* (*χ²*_*(1)*_ = 16.0; *P* < 0.001) and for *E. concordis* (*χ²*_*(1)*_ = 17.64; *P* < 0.001) (Fig. 4).

## Discussion

The present study showed that the oviposition rate of *A. largoensis* and *E. concordis* was influenced by the diet offered. For both predators, high egg production was observed when they were fed exclusively on *R. communis* pollen, at approximately 2 eggs/female/day (average of 3 days of observation). However, the availability of this resource did not reduce the consumption of *A. cocois* eggs by either species. The age of *A. cocois* eggs influenced predation by *A. largoensis* and *E. concordis*. Both species showed higher consumption of older eggs, a result observed in both choice and non-choice tests; however, prey age eggs did not affect the oviposition rate of these predators.

Generalist predatory mites are capable of feeding on a wide variety of food sources (Mcmurtry and Rodrigues 1987; van Rijn and Tanigoshi 1999; Abdallah et al. 2001; Nomikou et al. 2003c; Vantornhout et al. 2005; Calabuig et al. 2018; Etienne et al. 2021; Saito and Brownbridge 2021, 2022). These mites actively seek different food sources, diversifying their diet to balance their nutrient intake (Marques et al. 2015). As expected, *A. largoensis* and *E. concordis* consumed the different diets offered. A diet consisting exclusively of *A. cocois* eggs resulted in an increase in the average daily oviposition of these predators, when compared to a diet consisting exclusively of *T. urticae*. Since *T. urticae* is the most used prey in phytoseiid mite predation studies (see Akyazi et al. 2018; Samaras et al. 2019; Gravandian et al. 2022; Domingos et al. 2025), the result of feeding *E. concordis* and *A. largoensis* females with *A. cocois* eggs proved to be interesting. However, it is important to highlight that the experimental units for the different prey were constructed using different leaves (considering the host suitability of each prey). The characteristics of the leaves can cause different levels of stress in the predatory mites (Schmidt 2014), which may interfere with the egg production response (Gong et al. 2026). Therefore, this comparison needs to consider these differences, and further studies are needed to observe the effect of different leaves on oviposition of *E. concordis* and *A. largoensis*.

A diet of *A. cocois* eggs supplemented with castor bean pollen increased the reproductive potential of *E. concordis*, but not of *A. largoensis*, compared to a diet consisting exclusively of *A. cocois* eggs. This result can be explained by the well-documented preference of species from the genus *Euseius* spp. for pollen (Moraes and Mcmurtry 1978; Congdon and Mcmurtry 1988; Mcmurtry and Croft 1997; Furtado and Moraes 1998; Mcmurtry et al. 2013; Adar et al. 2014). Exclusive feeding of *A. cococis* eggs allowed the survival and development of *E. concordis* and *A. largoensis*; however, resulted in a low number of *E. concordis* females being able to oviposit with this type of diet (Alfaia et al. 2018a, b), corroborating our result. Thus, when offered alone, *A. cocois* eggs do not constitute the best diet option for *E. concordis*. However, the oviposition rate may indicate a more complex response. Predatory mites can adapt egg production to the surrounding environment. In the presence of stress conditions and low food quantity and/or quality, mites can balance the investment of resources that would be destined for oviposition in order to prolong their longevity (Gotoh and Tsuschiya 2009); furthermore, variations in food resources available to offspring can lead to the female making a greater investment of nutrients in individual eggs, rather than an increased oviposition rate (Gong et al. 2026). Therefore, the increase in the number of *E. concordis* eggs in the treatments with castor bean pollen supplementation may be a direct consequence of food availability in the experimental unit and not related to the nutritional quality of *A. cocois* eggs for this predator. However, this would not justify the increased oviposition of *E. concordis* and *A. largoensis* on diets consisting solely of castor bean pollen; thus, offering only *A. cocois* eggs proves a suboptimal diet for *E. concordis* and *A. largoensis*.

Supplementing the diet of predatory mites with pollen is already well known (Samaras et al. 2019; Urbaneja-Bernat and Jaques 2020; Barzka et al. 2023), and studies have demonstrated that pollen constitutes an adequate food source for the development and reproduction of phytoseiid mites (van Rijn and Tanigoshi 1999; Nomikou et al. 2003a; Gnanvossou et al. 2005; Rodríguez-Cruz et al. 2013; Duarte et al. 2015); however, not all types of pollen are suitable for them. Pollen can contain secondary metabolites produced by the plants to defend themselves against herbivores, since this is the male gamete, vital plant tissue (Palmer-Young et al. 2019; Stevenson 2020). Furthermore, the pollen of different plants exhibits great variation in their morphology (e.g., presence of spines), which can hinder its handling and interfere with the biology of predatory mites (Eini et al. 2022). Despite this, pollen is also an important source of nutritional elements, such as proteins, carbohydrates, lipids, vitamins, and minerals (Lau et al. 2022). Diversifying food consumption can be beneficial by helping to dilute toxins (Toft and Wise 1999) or correct specific nutritional imbalances (Mayntz et al. 2005). Therefore, the use of pollen from different plants has been extensively investigated regarding the improvement of the fitness of predatory mites (Goleva and Zebitz 2013; Kumar et al. 2014; Eini et al. 2022), even when a mixed diet with unsuitable prey is offered (Samaras et al. 2019).

Castor bean (*Ricinus communis*) pollen has been widely used as a food source in laboratory rearing of phytoseiid mites due to its high nutritional value and availability, often promoting increased oviposition and population growth in several species (Galvão et al. 2007; Marafeli et al. 2014). Its suitability as an alternative food resource has been demonstrated for generalist predators, particularly those capable of exploiting both prey and plant-derived resources, reinforcing its potential role in supporting predator populations in agroecosystems. This evidence supports the potential use of R. communis as a functional resource in agroecosystems aimed at enhancing the persistence and effectiveness of predatory mites.

Cattail (*Typha* sp.) and chilli pepper (*Capsicum frutescens*) pollen promoted higher oviposition in *Amblyseius herbicolus* (Chant) (Mesostigmata: Phytoseiidae) compared with exclusive feeding on *T. urticae* (Duarte et al. 2015). Similarly, offering *A. largoensis* an exclusive diet of prey (*Aceria guerreronis* Keifer or *T. urticae*) resulted in lower oviposition compared to a mixture of prey and castor bean (*R. communis*) pollen (Galvão et al. 2007). Therefore, the combination of nutrients from different food sources may provide the ideal scenario for increasing fitness in the predatory mites.

*Euseius concordis and A. largoensis* show potential for use in the control of *A. cocois*, particularly through egg consumption at low pest population densities (Alfaia et al. 2018a, b; Mendes et al. 2021). Supplying castor bean pollen or creating reservoir zones with castor bean plants in cashew-producing areas appears to be an interesting strategy for managing *A. cocois*, as it allows the maintenance of *E. concordis* or *A. largoensis* even when the presence of pest eggs is scarce. The use of non-cultivated plants (*Inga edulis*, *Senna macranthera*, and *Varronia curassavica*) as a reservoir for predatory mites was studied in intercropping with coffee (*Coffea arabica*) increasing the number of predatory mites in the production area (Ferla et al. 2023). Despite the positive relationship between pollen-producing plants and phytoseiid mites in production areas, the effect of intercropping with non-cultivated plants on pest control is not well established (Tixier 2018). Therefore, studies on the compatibility of cashew trees with castor beans plants, the dispersal of *E. concordis* and *A. largoensis* among these plants, and the population dynamics between these predator populations and *A. cocois* should be carried out to assess the possibility of intercropping use.

The presence of castor bean pollen does not make *A. cocois* eggs less attractive to *E. concordis* and *A. largoensis*, such that offering a mixed diet with castor bean pollen did not reduce the consumption of *(A) cocois* eggs by these predators. Therefore, the regulation of this pest density is not compromised by the availability of castor bean pollen, an important aspect for the effectiveness of biological control (Nomikou et al. 2003b, c). The addition of *Typha* sp. pollen to the diet with 1st instar nymphs of *(B) tabaci* did not alter the amount of prey consumed by the predatory mite *Typhlodromips swirskii* (Athias-Henriot) (Acari: Phytoseiidae), but it did affect the consumption of the predator *Euseius scutalis* (Athias-Henriot) (Acari: Phytoseiidae) (Nomikou et al. 2004). Feeding *Amblyseius swirskii* diets containing the prey *T. urticae* supplemented with pollen from different plants showed that the effect on predation rate depends on the type of pollen offered (Riahi et al. 2017). Furthermore, prey consumption of *N. californicus* in the presence of pollen is lower but compensated by higher fecundity and survival rates (Khanamani et al. 2017). These results reveal that the relationship between pollen supplementation and prey consumption rate is likely specific and does not follow a general pattern; therefore, it should be studied on a case-by-case basis.

Both *E. concordis* and *A. largoensis* showed a preference for older *(A) cocois* eggs (> 48 h old), consuming more eggs of this category than the younger ones (≤ 24 h old), even when offered together or separately in the experimental unit. *Amblyseius swirskii* and *N. californicus* have also shown higher consumption of older *T. urticae* eggs (Akyazi et al. 2018). Consumption of *T. urticae* eggs < 24 h old did not allow the predatory mite *N. barkeri* to reach the protonymph stage (Momen and Hussein 2011). In contrast, *(B) mali* exhibited higher consumption rates of younger *D. melanogaster* eggs (< 8 h old) (Michalska et al. 2023). In the case of predation of *B. tabaci* (biotype B) eggs by the predatory mite *(A) swirskii*, an interesting result was observed; in the choice test, no difference was observed between the predation of young (0–24 h old) and old (> 48 h old) eggs; in the no-choice test, the mites consumed a greater number of young *(B) tabaci* eggs (Cavalcante et al. 2015). These contrasting results indicate specificity in the relationship between prey egg age and predation preference, providing support for interpretations based on optimal foraging theory and adaptive responses associated with feeding preferences (Pyke 1984). In the first part of our study, the age of the *A. cocois* eggs was not standardized, and distribution occurred randomly among the diets, because, in the field *E. concordis* and *A. largoensis* will encounter *A. cocois* eggs of varying ages. The preference for very young eggs (< 24 h old) likely results in a shorter foraging time, where the predator must consume a greater number of eggs in a shorter period. Therefore, knowledge of predator preferences for different egg ages is important for predicting periods during which predatory mites exhibit greater predation efficiency across prey stages. In this context, Alfaia et al. (2018a), by evaluating functional response of *E. concordis* and *A. largoensis* on eggs with a maximum development time of 24 h, may have obtained responses different from those observed when older eggs are considered.

Understanding predation behaviors of mites and their reproductive responses is essential for designing integrated pest management strategies, allowing optimization of predator performance at the most susceptible stages of the pest and, consequently, reducing crop damage. We suggest further studies such as: evaluating strategies for the use of castor bean pollen in cashew production areas aiming at the control of *A. cocois*; analyzing ecological parameters of *E. concordis* and *A. largoensis* fed with old eggs of *A. cocois*.

## Data Availability

The raw data that support the findings of this study have been deposited in Zenodo and are publicly available at: **10.5281/zenodo.18425070**.
